# An Unusual Outcome Using the TightRope® System in Syndesmotic Injury Management: A Case Report

**DOI:** 10.7759/cureus.78634

**Published:** 2025-02-06

**Authors:** Fernando A Fernández-Garza, Omar F Rodríguez-Rodríguez, David Muñoz-Leija, Rodrigo E Elizondo-Omaña, Alejandro Quiroga-Garza, Felix Vilchez-Cavazos, Abraham G Espinosa-Uribe

**Affiliations:** 1 Traumatology and Orthopedics Department, Instituto de Seguridad Social de Trabajadores del Estado de Nuevo León (ISSSTELEON), Monterrey, MEX; 2 Traumatology and Orthopedics Department, Instituto de Seguridad y Servicios Sociales de los Trabajadores del Estado (ISSSTE) Hospital Regional, Monterrey, MEX; 3 Human Anatomy Department, Universidad Autónoma de Nuevo León, School of Medicine, Monterrey, MEX; 4 General Surgery Department, Instituto Mexicano del Seguro Social, Monterrey, MEX; 5 Orthopedics and Traumatology Service, Hospital Universitario “Dr. José Eleuterio González”, Universidad Autónoma de Nuevo León, Monterrey, MEX

**Keywords:** ankle fracture management, dynamic fixation, post-operative review ankle fracture, syndesmotic injury, tightrope fixation

## Abstract

Syndesmotic injuries of the ankle are critical for maintaining joint stability and function, especially during weight-bearing activities. While traditional management involves rigid fixation with trans-syndesmotic screws, limitations such as restricted micromovement, potential screw breakage, and the need for secondary procedures have spurred the adoption of dynamic fixation methods like the TightRope^®^ system (Arthrex, Naples, Florida, US). This report presents a case of a 54-year-old female with complications related to TightRope fixation following open reduction and internal fixation (ORIF) of a bimalleolar fracture. The case highlights the clinical course, surgical management, and outcomes, providing valuable insights into the limitations and potential complications of this technique.

## Introduction

The tibiofibular syndesmosis is essential for ankle stability, especially during weight-bearing activities. Injuries to this structure can result in significant functional impairment and long-term complications if improperly addressed. Syndesmotic injuries often occur alongside ankle fractures but can also present as isolated ligamentous injuries. These types of fractures account for about 10% of foot and ankle fractures [1,2].

Traditional management has relied on rigid fixation with trans-syndesmotic screws. However, these screws have limitations, including compromised joint biomechanics, screw breakage, malreduction, and stiffness, often necessitating secondary procedures to restore natural tibiofibular movement [3].

The TightRope® system from Arthrex (Naples, Florida, US) offers a less invasive, physiologically adaptive alternative. Utilizing high-strength sutures and endobuttons, it provides stable yet dynamic fixation, allowing the micromovement necessary for proper ankle function. While early studies report improved outcomes with the TightRope system, including better syndesmotic reduction and preserved mobility, complications can still arise [4,5].

This suture and endobutton system has been continuously studied, and multiple reviews show that it is a better alternative to conventional reduction with screws in many outcomes like less implant removal, better fracture reduction, and fewer postoperative complications [3,6-9].

This case report explores a rare reaction to the TightRope system, which relates to a need for implant removal.

## Case presentation

**Figure 1:**
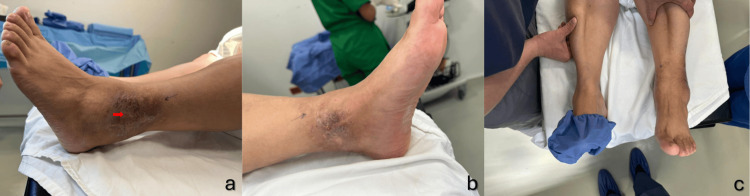
Visual comparative of the ankle a. Lateral view of the left ankle, displaying a fibrous atrophic surgical scar, with a red arrow pointing to the site of non-foul-smelling discharge. b. Medial view of the left ankle, showing heterochromia with irregular, non-elevated borders, and no evidence of hypertrophy or keloid scarring. c. Comparative view of both ankles, highlighting the increased volume in the left ankle.

A 54-year-old female patient presented to the clinic with whitish discharge from a surgical wound and edema related to an open reduction internal fixation (ORIF) procedure for a left bimalleolar ankle fracture performed two years ago. Personal medical history: Denied any chronic illnesses, allergies, or prior transfusions. Surgical history: Bilateral lacrimal duct opening and ORIF for a left trimalleolar ankle fracture. Current medications: Omeprazole 20 mg every 24 hours. Denies alcohol consumption, smoking, drug use, or taking any type of supplementation. The patient reported persistent whitish, non-smelling discharge (Figure 1).

**Figure 2:**
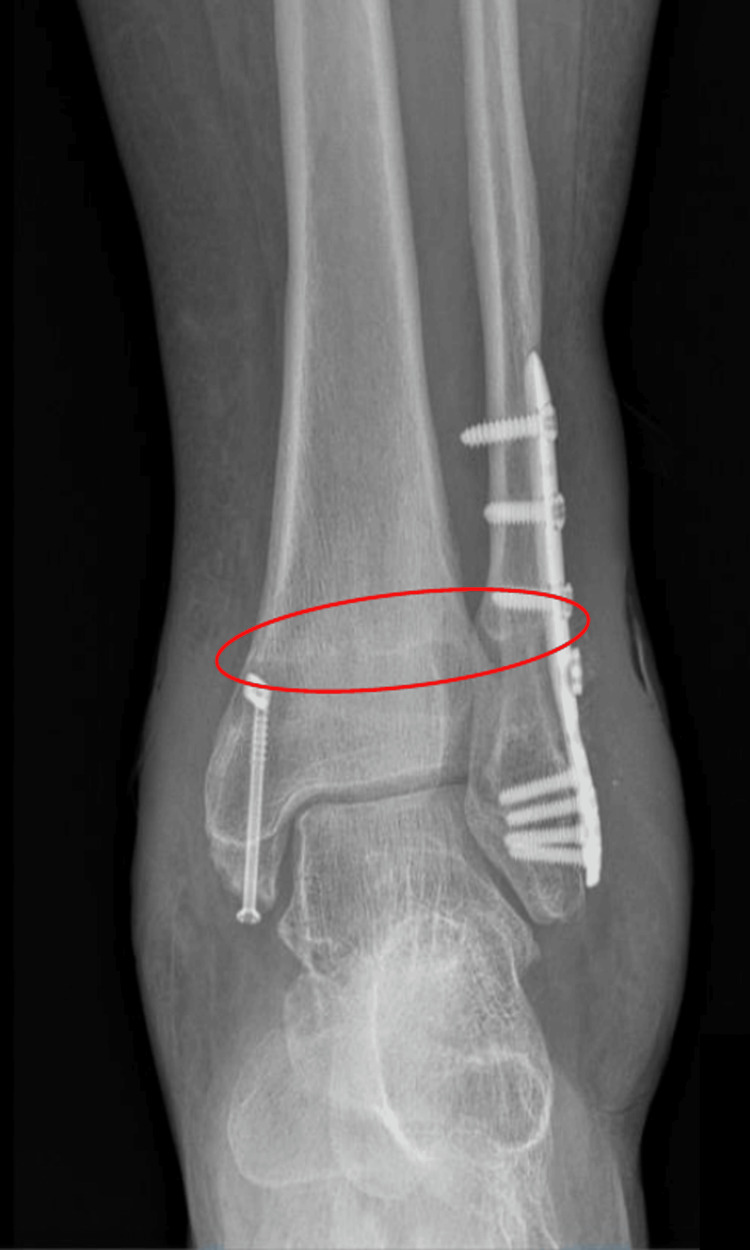
Anteroposterior X-ray of the ankle showing proper alignment with the presence of osteosynthesis material A radiolucent line corresponding to the course of the TightRope is visible (red circle).

Upon inquiry, she confirmed maintaining daily hygiene. On physical examination, the surgical scar appeared well-aligned and hydrated, with mild whitish discharge and no tenderness upon palpation. Anteroposterior (AP) and lateral radiographs of the left leg showed articular congruence and proper positioning of the anatomical fibular plate without signs of lysis. Radiolucency was noted at the insertion site of the TightRope and the medial malleolus cannulated screw (Figure 2).

Laboratory results are shown in Table 1.

**Table 1:** Laboratory results PT, Prothrombin Time; PTT, Partial Thromboplastin Time; INR, International Normalized Ratio; BUN, Blood Urea Nitrogen

Parameter	Results	Reference value
Hemoglobin	13.2 g / dL	12.20 – 18.10
Hematocrit	41.8%	37.7 – 53.7
Platelets	285,000 K / uL	142.00 – 424.00
Leukocytes	7,880 K / uL	4.00 – 11.00
PT	12.1 s	10.68 – 12.08
PTT	36.6 s	28.0 – 35.6
INR	1.02	
Glucose	105 mg/dL	60 - 100
Urea	19 mg/dL	12.8 – 42.8
BUN	8 mg/dL	7 - 20
Creatinine	0.78 mg/dL	0.6 – 1.4

Management 

The patient was initially prescribed a topical cream containing acexamic acid, neomycin, and zinc oxide to promote healing. However, there were no favorable results due to continued secretion from the surgical wound. A surgical procedure was planned for the removal of the TightRope system, along with the collection of tibial and fibular endosteal cultures and osteosynthesis material. Both bacterial culture and potassium hydroxide (KOH) tests yielded negative results.

Follow-up

**Figure 3:**
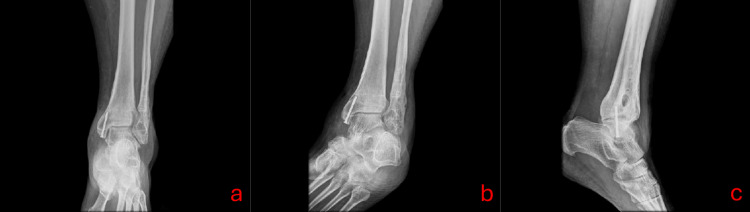
Anteroposterior (a), oblique (b), and lateral (c) radiographs of the left ankle. Bony alterations are observed in the distal tibiofibular syndesmosis region and the areas corresponding to the previous osteosynthesis material. Radiolucent tubular images with well-defined edges are identified, consistent with the previous TightRope pathway. Peripheral sclerosis is noted at the margins of the tunnels, without evidence of active lysis or osteolytic reaction.

At the 10-day follow-up, the patient reported mild pain. Examination revealed a healing scab with no signs of infection. At six weeks post-removal, the surgical site exhibited a well-formed scar with subcutaneous fibrosis and no drainage. A control radiograph was taken and is shown in Figure 3.

The patient demonstrated satisfactory healing and resolution of symptoms. Twelve weeks after the removal of the material, the patient was physically active in work-related activities.

## Discussion

The TightRope system represents an innovative approach to managing syndesmotic injuries. In this case, while the TightRope system initially provided adequate stabilization, delayed complications required additional intervention. The successful resolution following system removal suggests that timely recognition and management of complications are crucial for optimal outcomes.

This case report presents a scenario of persistent wound discharge and implant-related issues following TightRope fixation for a syndesmotic injury. Hong et al. reported an adverse event associated with the use of the TightRope system [10]. Similarly, they described a non-healing wound with discharge, which healed appropriately after the removal of the implant. In contrast to our case, an external fixation with internal reduction in a first plane was not performed. The authors noted that the TightRope knot was overly prominent and was even cut multiple times. Additionally, there was a shift in the position of the osteosynthesis material, and upon removal of the TightRope, cultures tested positive for methicillin-sensitive Staphylococcus aureus, in addition exposure and manipulation of the material may be considered a risk factor for implant infection.

This suture-button device is normally well-tolerated, and many studies have shown that the rate of implant removal or implant failure is significantly lower in this type of system than in conventional screws [9]. In our experience, we have used this device on multiple occasions, with this being the first time we encounter a case where it needs to be removed. It is worth noting that cultures taken from the material extraction were negative.

Previous studies have reported complications associated with the TightRope system, including skin irritation, implant prominence, and persistent pain [1,9,11]. Although it is expected that there will be no need to remove the device, a systematic review found a removal prevalence ranging from 0% to 13.3%, with an overall rate of 3.7%. The main reason for device removal was implant irritation [9]. Storey et al. reported 102 cases using the TightRope system, but only 31 involved Weber B fractures, like our patient’s [12]. The study found higher fracture reduction rates and fewer complications in Weber B cases. Of the 14 reported complications, 10 occurred in Weber C fractures, suggesting higher mechanical demands in these cases. Additionally, the study does not differentiate complications by fracture type, which is crucial for treatment selection.

However, the delayed onset of wound issues in our case is a noteworthy observation. Even with this complication, the TightRope system has some advantages like dynamic stabilization, less surgical invasiveness, and preservation of joint biomechanics [4,7].

An important characteristic in this case described is that the patient did not have any comorbidity that could condition the removal of the implant such as immunodeficiencies, a history of removal of similar material used in the TightRope system, and others. In this patient, the only resolution of this eventuality was the removal of material, being that this system has one of the lowest rates of implant removal, malreduction, or complications [4,7,9].

## Conclusions

The TightRope system represents a valuable tool in the management of syndesmotic injuries. However, surgeons should be aware of the potential for complications, especially in challenging cases. Careful patient selection, meticulous surgical technique, and vigilant postoperative monitoring are essential to optimize outcomes and minimize adverse events. Continued research and refinement of surgical techniques are necessary to optimize outcomes for patients with syndesmotic injuries.
